# Ataxia Oculomotor Apraxia Type 1 in the Siblings of a Family: A Novel Mutation

**Published:** 2017

**Authors:** Parvaneh KARIMZADEH, Simin KHAYATZADEH KAKHKI, Shaghayegh Sadat ESMAIL NEJAD, Masood HOUSHMAND, Mohammad GHOFRANI

**Affiliations:** 1 Pediatric Neurology Research Center, Shahid Beheshti University of Medical Sciences, Tehran, Iran; 2 Pediatric Neurology Department, Mofid Children’s Hospital, Faculty of Medicine, Shahid Beheshti University of Medical Sciences, Tehran, Iran; 3 Pediatric Department, Shahid Beheshti University of Medical Science,Tehran, Iran; 4 Department of Medical Genetic, National Institute for Genetic Engineering and Biotechnology(NIGEB), Tehran, Iran

**Keywords:** Ataxia-oculomotor apraxia, AOA1, APTX gene, new mutation

## Abstract

Although AOA1 (ataxia oculomotor apraxia1) is one of the most common causes of autosomal recessive cerebellar ataxias in Japanese population, it is reported from all over the world. The clinical manifestations are similar to ataxia telangiectasia in which non-neurological manifestations are absent and include almost 10% of autosomal recessive cerebellar ataxias. Dysarthria and gait disorder are the most two common and typical manifestations. Oculomotor apraxia is usually seen a few years after the manifestations start. APTX gene on 9p13.3 chromosome is expressed in the cells of all human body tissues and different mutations had been discovered. Here we report two siblings (a girl and a boy) of consanguineous parents visited at Mofid Pediatrics Hospital in 2015, with history of gait ataxia, titubation, tremor, and oculomotor apraxia around five yr old and after that. The brother showed symptoms of disease earlier and more severe than his sister did. After ruling out the common etiologies of progressive ataxia, we did genetic study for AOA1 that showed a homozygous frameshift mutation as c.418_418 del was found. This mutation was not reported before so this was a new mutation in APTX gene.

## Introduction

Ataxia with oculomotor apraxia type1 (AOA1) is rare a cause of autosomal recessive cerebellar ataxia (ARC) manifested by progressive cerebellar ataxia, severe peripheral neuropathy, oculomotor apraxia, and hypoalbuminemia (1-3). The diagnosis is based on physical examination, absence of non-neurological signs, the progressive nature of disease and the positive familial history (2). 

The EMG findings are indicative of a severe sensorimotor axonal neuropathy. The oculographic records show” hypo metric saccades, decrease mean gain in amplitude, broken saccades into multiple successive saccades and normal latencies” (3). 

Brain MRI shows brain atrophy. Hypercholesterolemia and hypoalbuminemia are common (the duration of disease has reverse relation with albumin level and direct relation with cholesterol level). The diagnosis is confirmed by molecular analysis of APTX gene (3). The differential diagnosis are ataxia telangiectasia, disorders similar to ataxia telangiectasia, friedrich ataxia, AOA2, ataxia with vitamin E deficiency and spastic AR ataxia Charlevoix-Saguenay. Prenatal tests and carrier testing are available for family members if both alleles of disease are known in a family (4). 

There is no specific treatment for AOA1 and the treatment is based on supportive procedures including rehabilitation therapy of disabilities caused by peripheral neuropathy and cerebellar ataxia, educational supporting on reading and writing problems, and of course speech therapy of dysarthria and cognitive disorders. Low fat and low cholesterol diet are recommended. An expert neurologist should do routine follow-up. Some experimental therapies such as CoQ10 had been tried for assessing the effectiveness of treatment (1). 

Overall, AOA1 is a progressive neurodegenerative disorder and the patients usually lose motor skills and become wheel-chair bound 7-10 yr after the disease starts.

## Case report

Here we report a family with two affected children from consanguineous parents visited at Mofid Pediatrics Hospital in 2015. The clinical manifestation was compatible with AOA1. First (female) child was 17 yr old, showed presenting feature at about 5 yr old with ataxia, titubation, and dysarthria followed by ocular apraxia. In physical examination, she had ataxic gait, areflexia, titubation, supranuclear gaze palsy, and oculomotor apraxia. Another sibling was a boy that showed the same symptoms, but earlier and more severe. Informed consent was obtained from the parents for publication of the case report. 

In their family history their aunt (mother’s sister) had had this problem; in physical examination, prominent finding was ataxic gait, hyporeflexia, downward plantar reflex, titubation, oculomotor apraxia, and supranuclear gaze palsy. The mental assessment was normal. 

Evaluation for ataxia was done: CBC, TG, Chol, Alb, AFP, CEA, CPK, LDH, Aldolase, Ceruloplasmin, ammonia lactate, pyruvate, liver function test, HPLC, electrophoresis of hemoglobin, vit B12 & vit E level was normal. EMG, NCV showed the sign of sensory peripheral neuropathy; MRI showed normal finding. The genetic study for SCA1, 2, 3, 6, 7 was normal. Because of SNGP and ataxia, genetic study for NPC 1, 2 was performed which was negative. At the last genetic study for AOA1 showed a new homozygous frameshift mutation in this family.

## Discussion

AOA1 is caused by a mutation of APTX gene (9p13.3) which codes aprataxin and its role is DNA repairment (1, 2). Most of the mutations are in fifth, sixth and seventh exons. A relation between the genotype and phenotype of the disease is proven. For example, severe choreic form phenotype is seen in A198V mutation. Truncating mutation is accompanied by early onset disease and severe intellectual disability phenotype. 

Aprataxin is produced in different human body tissue such as muscles, spinal cord, brain and cellular nucleus. Different parts of aprataxin repair DNA and RNA. It binds to DNA and RNA and makes molecular reactions with other repairing proteins. In the site of damage, aprataxin changes the damaged ends of DNA and RNA and helps them to reconnect. 

At least, 30 different mutations causing AOA1 had been seen in APTX gene, which most of them make a single change in protein structure, block an amino acid in aprataxin and produce an abnormal rapid-destroyable protein, therefore, the DNA repairing defects (3,4). This causes DNA cell accumulation especially in cerebral and cerebellar cells, and such accumulations make cell apoptosis accelerated and this is specified for motor disorders of AOA1(3).

AOA1 is almost 3.6% of all ARCA cases in Portugal (4), but it is the most common cause of ARCA in Japan. In a cohort study, 227 mostly French patients (158 families) with progressive cerebellar ataxia (after Friedrich ataxia ruled out) were done in which AOA1 frequency was almost 5% (4). In this study, a large group of patients were studied with 158 families with non-Friedrich progressive ataxia Of 14 cases (9 families) had been shown five different types of missense or truncating mutations of aprataxin (A198V, d267G, W279X, W279R, IVS51). Howover, four new mutations were reported. The frequency of AOA1 in this study was 5%. The mutation careers were assessed in different aspects of disease: neurological, neuropsychological, electrophysiological, oculographic, biologic, MRI findings and physical examination (3). 

The disease onset age was 6.8±4.8 (2-18 yr) (3). Cerebellar ataxia, atrophy in MRI with severe axonal and sensorimotor neuropathy were reported in all patients, but hypercholesterolemia (75%), hypoalbuminemia (83%) and oculomotor apraxia (86%) were seen variable and were not reported in all patients. The chorea was common in the onset of disease (79%), but it disappeared. The severe and permanent phenotype of chorea was reported with one of a special type of mutation (A198V) (3). 

In our research, the onset age of the disease was 5 yr in sisters and 3 yr in brothers of the patients, which was consistent with the age spectrum of AOA1. Ataxia and oculomotor apraxia were seen in both siblings. Hypercholesterolemia and hypoalbuminemia were not reported. The chorea was more obvious in younger patients and cognitive disorders were seen in both siblings, however, they were more severe in male siblings. 

A male patient was reported with AOA1 who was homozygotes in G837A, W279X) mutation of APTX. The manifestations were seen in 3.5 yr old that were atypical: absence of oculomotor apraxia, chorea, and cerebellar atrophy. Peripheral neuropathy was shown in 8 yr old. Cerebellar atrophy was not seen in our study even in older patients (5). 

A clinical heterogeneity was found in the study in patients with early onset ataxia in accompanying by hypoalbuminemia). EAOH is a variant of Friedrich ataxia in Japan. In the progression of disease, the severity of chorea-like movements was decreased and hypoalbuminemia became more obvious. Molecular analysis showed two missense mutations, one insertion mutation and one new missense mutation (6). 

A frame shift mutation was reported that had more severe phenotype than missense mutations. The relation of genotype with early onset ataxia, oculomotor apraxia and hypoalbuminemia were under a disagreement and challenging atmosphere. Although, frame shift mutations were seen in our patients, signs and symptoms were not consistent with the genotype mentioned in previous studies and it was less severe (7). 

Hypoalbuminemia were seen in 5 Portuguese patients during their disease follow-up and they suggested that EOCA-HA and AOA1 were same diseases. We have seen no hypoalbuminemia and hypercholesterolemia during the follow-up of our patients (8). 

Totally, 28 Italian patients were studied who had peripheral neuropathy and oculomotor apraxia. Three of them had APTX mutation and had AOA1. H201Q (a new missense homozygote mutation) in one patient and P206L (a Japanese missense mutation) in two patients were reported. Heterogeneity in clinical manifestations and late onset of disease in comparison of other reports were seen in this study (9). 

A missense mutation can lead to a late onset of clinical manifestations (almost in 9 yr old) nut frameshift or nonsense mutation had an onset of 2-12 yr (mean age: 4.96 yr). Besides, a relation between the mutation and the severity of cognitive disorders, dystonia, and chorea was found. Cerebellar ataxia, peripheral neuropathy, and cerebellar atrophy were most common manifestations. Therefore, a new missense mutation in their patients was reported (10). 

**In Conclusion,** different clinical phenotypes and different multiple mutations had been already found. Therefore, in all cases of oculomotor involvements with cerebellar ataxia, the diagnosis of AOA1 should be kept in mind. Our patients did not have hypoalbuminemia and hypercholesterolemia. The MRI was normal. The onset age of disease was earlier in frameshift mutations coordinated with previous studies but the severity was not similar to them. The female patient was 17 yr old and was not wheelchair bound yet, but the male patient had more severe manifestations and the progression should be followed up. They also had new mutations not reported already.

## Author Contribution

Parvaneh Karimzadeh: Evaluating and treating the patients, designing the work, supervising and editing the article. 

Simin Khayatzadeh Kakhki: Corresponding author, drafting, analysis, and writing the manuscript. Masoud Houshmand: Performing molecular analysis.

 Shaghayegh Sadat Esmail Nejad: Drafting.

Mohammad Ghofrani: Supervising. 

All authors agreed to be accountable for all aspects of the work in ensuring that questions related to the accuracy or integrity of any part of the work are appropriately investigated and resolved.

## References

[1] Jafar-Nejad P, Maririch SM, Zoghbi HM, Swaiman KF, Ashwal S, Ferriero DM, Schor NF (2012). The cerebellum and hereditary ataxias. Swaiman’s Pediatric Neurology Principles, and Practice.

[2] Pina-Garza JE, Pina-Garza JE (2013). Ataxias. Fenichel Clinical pediatric neurology.

[3] Le Ber I, Moreira MC, Rivaud-Péchoux S, Chamayou C, Ochsner F, Kuntzer T (2003). Cerebellar ataxia with oculomotor apraxia type 1: clinical and genetic studies. Brain.

[4] Coutinho P, Barbot C, Pagon RA, Adam MP, Ardinger HH (1993-2016). Ataxia with Oculomotor Apraxia Type 1. 2002 Jun 11. GeneReviews® [Internet].

[5] Shahwan A, Byrd PJ, Taylor AM, Nestor T, Ryan S, King MD (2006 ). Atypical presentation of ataxia-oculomotor apraxia type 1. Dev Med Child Neurol.

[6] Shimazaki H, Takiyama Y, Sakoe K, Ikeguchi K, Niijima K (2002). Early-onset ataxia with ocular motor apraxia and hypoalbuminemia: the aprataxin gene mutations. Neurology.

[7] Yokoseki A, Ishihara T, Koyama A, Shiga A, Yamada M, Suzuki C, and et al (2011). Genotype-phenotype correlations in early onset ataxia with ocular motor apraxia and hypoalbuminaemia. Brain.

[8] Moreira MC, Barbot C, Tachi N, Kozuka N, Mendonça P, Barros J, Coutinho P, Sequeiros J, Koenig M (2001). Homozygosity mapping of Portuguese and Japanese forms of ataxia-oculomotor apraxia to 9p13, and evidence for genetic heterogeneity. Am J Hum Genet.

[9] Criscuolo C, Mancini P, Saccà F, De Michele G, Monticelli A, Santoro L, Scarano V, Banfi S, Filla A (2004). Ataxia with oculomotor apraxia type 1 in Southern Italy: late onset and variable phenotype. Neurology.

[10] Houshmand M, Nouri Nay, Nouri Nar, Aryani Om (2012). A Novel Mutation in the Aprataxin (APTX) Gene in an Iranian Individual Suffering Early-Onset Ataxia with Oculomotor Apraxia: Type 1(AOA1) Disease. IBJ.

